# The Effects of Resistance Training on Pain, Strength, and Function in Osteoarthritis: Systematic Review and Meta-Analysis

**DOI:** 10.3390/jpm14121130

**Published:** 2024-11-30

**Authors:** Jaehyun Lim, Ahyoung Choi, Byeonggeun Kim

**Affiliations:** 1 Department of Physical Therapy, Graduate School, Nambu University, Gwangju 62271, Republic of Korea; 2 Department of Rehabilitation, Songwon University, Gwangju 61756, Republic of Korea; 3 Department of Physical Therapy, Nambu University, Gwangju 62271, Republic of Korea

**Keywords:** osteoarthritis, resistance training, function, pain, strength

## Abstract

**Background/Objectives**: Pain is the most common symptom of osteoarthritis (OA), and it leads to functional decline, such as decreased mobility and limitations in activities of daily living, which leads to difficulties in social participation, increased social isolation, and economic burden. Muscle weakness can be a cause of OA symptoms. The purpose was to analyze the effects of resistance training on improving pain, strength, and function in OA and to analyze the effects by intervention duration and joint. **Methods**: The study search was conducted on 14 September 2024, and the period of study inclusion covered studies available in the databases from their inception to the search date. The databases used were PubMed, CHINAL, Cochrane Library, and Embase. Inclusion criteria were studies that targeted OA and compared a resistance training intervention with a no resistance training intervention group and measured pain, strength, and function. Subgroup analysis was used to analyze the effects by intervention duration (4 weeks or less, 5 to 8 weeks, 9 weeks or more) and joint (knee, hip). **Results**: A total of 27 studies included 1712 subjects, and significant improvements were observed in pain (SMD: −0.48, CI: −0.58~−0.37, I^2^: 45%), strength (SMD: 0.4, CI: 0.32~0.47, I^2^: 0%), and function (SMD: −0.56, CI: −0.65~−0.47, I^2^: 30%). In the effects by intervention duration, both pain and strength showed significant improvements, but no effect on function was observed for less than 4 weeks. For effects by joint, both the knee and hip showed significant improvements. **Conclusions**: Resistance training was effective in improving pain, strength, and function in patients with knee and hip OA.

## 1. Introduction

Osteoarthritis (OA) is a synovial joint disease that affects 12–15% of people aged 25–74, and its prevalence increases with age, with more than 70% of people aged 65 and older showing radiographic signs [1]. OA causes swelling, limitation of joint function, pain, and stiffness, and affects mental health, sleep, work participation, and mortality [2]. Increased ligament stiffness, altered muscle activation patterns, and decreased muscle strength can adversely affect joint kinematics and lead to degenerative changes in cartilage [3]. Pain is the most common symptom of OA, and the fear of using the knee joint due to pain leads to the weakening of muscle strength and a decrease in quality of life [4]. In addition, functional decline, such as reduced mobility and limitations in activities of daily living, leads to difficulties in social participation, increased social isolation, and economic burden [5].

Pain in OA is exacerbated by various factors, including increased joint loading and systemic inflammation associated with obesity. For overweight or obese individuals, reducing body weight by 7.5% or more has been shown to lower pain severity and decrease the risk of joint replacement surgery [6]. The increasing number of replacement surgeries for arthritis of the hip and knee joints highlights the need for effective non-surgical treatments for OA [7]. Osteoarthritis Research Society International (OARSI) recommends strengthening, cardio, balance training, and neuromuscular exercise programs as core recommendations for non-surgical management of knee and hip OA [8]. Among these, in terms of muscle strength, if the muscle strength of the extensor and flexor muscles of the knee joint is low [9], the risk of OA worsening increases, and it was found that knee OA patients had weaker quadriceps muscle strength than healthy adults [10]. In addition, patients with hip OA were found to have weaker hip abductor and knee flexor muscle strength than healthy adults [11]. Therefore, low muscle strength may be a cause of OA symptoms, and restoring muscle strength may reduce the risk of OA [12].

The effects of supervised progressive resistance training on function, pain, and quality of life in hip OA [13], the effects of resistance training on gait velocity and knee adduction moment in knee OA [14], and the effects of resistance training on pain, stiffness, and physical function [15] have been investigated. Recently, a study was conducted to compare the effects of resistance exercise on hip and knee OA according to the type of comparison group and to analyze whether improvements in muscle strength were associated with improvements in pain and physical function [16].

To date, meta-analysis studies have been conducted to analyze the effects of resistance training in OA patients, but no studies have reported the effects on improving muscle strength. Therefore, the purpose of this systematic review and meta-analysis was to analyze the effects of resistance training on improving pain, strength, and function in OA and to analyze the effects by intervention duration and joint.

## 2. Materials and Methods

### 2.1. Procedure

The PROSPERO registration (CRD42024584142) was performed prior to this study. In addition, this systematic review and meta-analysis was conducted according to the guidelines of the PRISMA 2020 checklist [17].

### 2.2. Eligibility Criteria

The eligibility criteria for this study were determined and selected based on the PICOS criteria. Patient: Subjects diagnosed with OA of the knee and hip; Intervention: Studies that performed any type of resistance training were selected, and if the intervention method specifically stated that strength training was the main focus; Comparison: If the intervention program did not include strength training; Outcomes: All outcomes measuring pain, strength, patient-reported disability were included, and there were no restrictions on the outcome measures used; Study design: Randomized controlled trials. Exclusion criteria included studies that included patients with conditions or surgeries other than OA, studies with intervention protocols that included other training, such as balance or aerobic exercise, studies without pre- and post-intervention mean standard deviations, studies without peer-reviewed gray literature, studies without full text, and studies written in languages other than English.

### 2.3. Search Strategy

The study search was conducted on 14 September 2024, and the period of study inclusion covered studies available in the databases from their inception to the search date. The databases used were PubMed, CHINAL, Cochrane Library, and Embase, and the search terms were “Osteoarthritis” AND “Knee” OR “Hip” AND “Strength training” OR “Resistance training” to search for studies. The study selection was performed by one reviewer who used EndNote20 to read duplicate studies, titles, and abstracts, and excluded studies that were irrelevant to the present study. Afterward, the text was read to select studies that met the purpose of the present study (provided in Supplementary Table S1).

### 2.4. Data Collection

Data extraction was conducted by two reviewers. After comparing the extracted data, if there was a disagreement between the reviewers, it was resolved through discussion. If the disagreement could not be resolved, the final decision was made according to the co-author. The extracted data included the first author, year of publication, number of experimental and comparison groups, sex, age, body mass index (BMI), target joint, radiographic diagnosis grade, intervention method in the experimental and comparison groups, intervention duration, number of interventions, intervention time, measured variables, and pre- and post-intervention mean standard deviation values.

### 2.5. Quality Assessment

Study quality was assessed using the Physiotherapy Evidence Database (PEDro) scale. The PEDro scale consists of 11 items, and items 2 to 11 were evaluated, excluding item 1, which affects external validity and is not included in the score. The evaluation contents include random allocation, blinding (allocation, patient, therapist, assessor), intention to treat, and statistical analysis. The classification criteria for the evaluation results are 0 to 3 points as poor, 4 to 5 points as fair, 6 to 8 points as good, and 9 to 10 points as excellent [18]. Two reviewers assessed the quality of the studies, and if the results of the assessment were inconsistent, a decision was reached by discussion. If the disagreement could not be resolved, it was resolved by the decision of the co-author.

### 2.6. Statistical Analysis

Statistical analysis was performed using RStudio to conduct a meta-analysis of the mean standard deviation values before and after the intervention. The collected outcome is a continuous variable and has various measurement methods, so the effect size was presented in a forest plot as standardized mean difference (SMD) and 95% confidence intervals (CI). The SMD was calculated using Hedges’ g as a random effects model because the participants and intervention methods of the included studies were diverse, and the effect size was interpreted as 0.2: small; 0.5: medium; 0.8: large [19]. In addition, the effects of resistance training by intervention duration (4 weeks or less, 5 to 8 weeks, 9 weeks or more) and joint (knee, hip) were analyzed by subgroup analysis. The effect size was expressed by matching pain and function with negative numbers and strength with positive numbers. In addition, among the Western Ontario and McMaster Universities arthritis index (WOMAC) items, pain was classified as pain and function, stiffness, and total were classified as function, and the effect size was analyzed.

Heterogeneity was assessed using the I^2^ statistic. The heterogeneity assessment criteria were set to 25% low, 50% medium, and 75% high [20]. Publication bias analysis was conducted to visually analyze whether the effect size was symmetric using a funnel plot. When asymmetry was found in the funnel plot, additional analysis was performed using Egger’s regression; when publication bias was confirmed, the effect size was reanalyzed after adjustment for error using trim-and-fill.

## 3. Results

### 3.1. Study Selection Process

**Figure 1:**
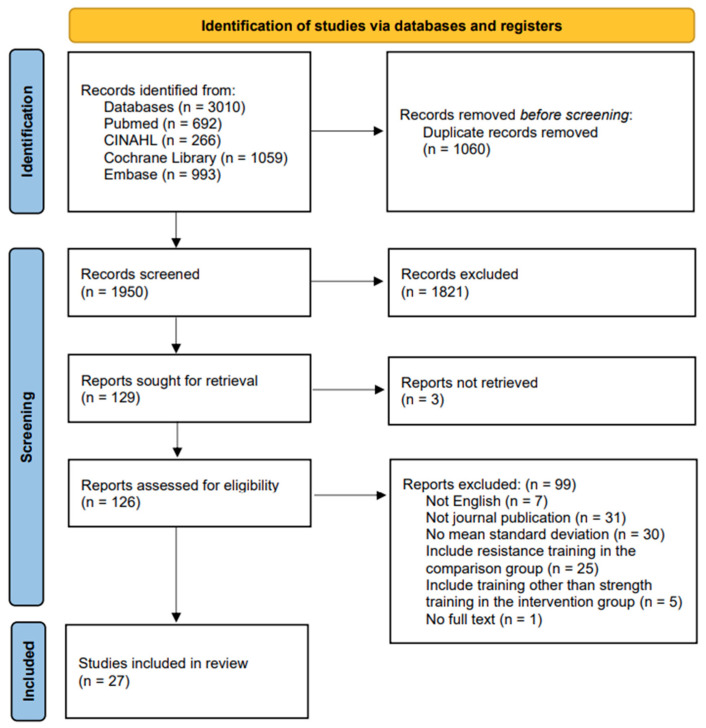
Flow diagram.

A total of 3010 studies were retrieved from Pubmed (n = 692), CINAHL (n = 266), Cochrane Library (n = 1059), and Embase (n = 993). After excluding 1060 duplicates, the remaining 1950 studies were screened by title and abstract to exclude 1821 studies that were not relevant to our study and 129 studies were selected. After screening the texts of the remaining 126 studies, excluding three studies that were not retrieved, 99 studies were excluded, and a final 27 studies were selected [21,22,23,24,25,26,27,28,29,30,31,32,33,34,35,36,37,38,39,40,41,42,43,44,45,46,47] (Figure 1). Tak et al. described a focus on strength training, but we excluded it because the training program included a treadmill [48].

### 3.2. Study Characteristics

The total number of subjects included in this review was 1712, including 955 in the experimental group and 757 in the control group. The average age of the participants in the included studies ranged from 53.8 ± 7.7 years in the group with the lowest average age to 71.05 ± 6.45 years in the group with the highest average age, while the average BMI ranged from 23.9 ± 4.60 in the group with the lowest BMI to 33.9 ± 8.3 in the group with the highest BMI. Of the 27 studies, 25 included knee OA and two included hip OA. Radiographic grading was used for both knees and hips using the Kellgren–Lawrence score, and all grades from grade 1 to grade 4 were included. Resistance training methods included training of the knee extensors in all studies, as well as training of the knee flexors, hip flexors/extensors/abductors/adductors, trunk, etc. Intervention duration ranged from 8 weeks to 18 months, and intervention frequency ranged from one to three times per week. The outcomes assessed in this review included pain, functional status, and muscle strength. Pain outcomes were assessed using the Visual Analog Scale (VAS), the WOMAC pain subscale, and the intermittent and constant osteoarthritis pain (ICOAP) scale. The WOMAC pain subscale measures pain during specific functional activities, providing a multidimensional perspective, while the VAS captures overall pain intensity on a single-dimensional scale. The ICOAP scale evaluates both intermittent and constant pain, offering insights into the temporal characteristics of osteoarthritis pain. This combination of tools allowed for a more comprehensive assessment of pain across studies, capturing specific activity-related pain, overall pain experiences, and the variability of pain over time. Functional outcomes were assessed using the Western Ontario and McMaster Universities Osteoarthritis Index (WOMAC), the Hip Disability and Osteoarthritis Outcome Score (HOOS), the Knee Injury and Osteoarthritis Outcome Score (KOOS), the 36-item Short Form Survey (SF-36), and activities of daily living (ADL) measures. Muscle strength outcomes were assessed by measuring knee or hip muscle torque and power (provided in Supplementary Table S2).

### 3.3. Quality Assessment

As a result of the quality assessment of the studies using the PEDro scale, a total of seven studies were rated as fair (one study with 4 points, six studies with 5 points) and a total of 20 studies were rated as good (nine studies with 6 points, nine studies with 7 points, and two studies with 8 points) (provided in Supplementary Table S3). All studies were randomized, similar at baseline, and provided outcome comparisons and measurements. In addition, 12 cases of allocation concealment, three cases of participants blinding, two cases of therapists blinding, 15 cases of assessors blinding, 16 cases of obtaining results from 85% of participants, and 11 cases of intention to treat were assessed.

### 3.4. Meta-Analysis Results

#### 3.4.1. Pain

Twenty-four studies were included in the analysis [21,22,24,25,26,27,28,29,30,31,32,33,34,35,37,38,39,40,41,42,43,45,46,47]. Analysis of the effect of resistance training on the pain of OA patients showed that it had a moderate effect on reducing pain compared to all groups (SMD: −0.48, CI: −0.58~−0.37, I^2^: 45%). The effect size by comparison group for resistance training showed a moderate effect on reducing pain compared to the no intervention group (SMD: −0.57, CI: −0.71~−0.44, I^2^: 8%). It was shown to have a moderate effect on reducing pain compared to the other intervention group (SMD: −0.42, CI: −0.59~−0.25, I^2^: 57%) and a moderate effect on reducing pain compared to the usual care group (SMD: −0.41, CI: −0.72~−0.1, I^2^: 32%). No differences were found between groups (*p* = 0.31) (provided in Supplementary Figure S1).

#### 3.4.2. Strength

Eleven studies were included in the analysis [23,24,26,28,30,32,34,36,38,39,44]. Analysis of the effect of resistance training on the strength of OA patients showed that it had a moderate effect on increasing strength compared to all groups (SMD: 0.4, CI: 0.32~0.47, I^2^: 0%). The effect size by comparison group for resistance training showed a moderate effect on increasing strength compared to the no intervention group (SMD: 0.45, CI: 0.36~−0.54, I^2^: 4%), and it was shown to have a moderate effect on increasing strength compared to the other intervention group (SMD: 0.31, CI: 0.19~0.43, I^2^: 0%). It was shown to have no effect on increasing strength compared to the usual care group (SMD: 0.39, CI: −0.01~0.79, I^2^: 0%). No differences were found between groups (*p* = 0.18) (provided in Supplementary Figure S2).

#### 3.4.3. Function

Seventeen studies were included in the analysis [24,25,27,28,30,31,32,33,34,35,38,39,40,41,42,46,47]. Analysis of the effect of resistance training on the function of OA patients showed that it had a moderate effect on reducing functional disability compared to all groups (SMD: −0.56, CI: −0.65~−0.47, I^2^: 30%). The effect size by comparison group for resistance training showed a moderate effect on reducing functional disability compared to the no intervention group (SMD: −0.74, CI: −0.91~−0.58, I^2^: 39%). It was shown to have a moderate effect on reducing functional disability compared to the other intervention group (SMD: −0.53, CI: −0.65~−0.42, I^2^: 0%), and a moderate effect on reducing functional disability compared to the usual care group (SMD: −0.34, CI: −0.48~−0.2, I^2^: 19%). Significant differences were found between groups (*p* = 0.01) (provided in Supplementary Figure S3).

#### 3.4.4. Subgroup Analysis

The effects of resistance training on OA by intervention duration and joint were analyzed by subgroup analysis (Table 1). Regarding the effect of intervention duration on pain, it was found to be effective in reducing pain for the duration of 4 weeks or less (SMD: −0.42 CI: −0.78~−0.06, I^2^: 38%), 5~8 weeks (SMD: −0.51 CI: −0.64~−0.38, I^2^: 0%), and 9 weeks or more (SMD: −0.47 CI: −0.64~−0.3, I^2^: 61%). There was no significant difference between the subgroups by the intervention duration (*p* = 0.95). Regarding joint-specific effects, it was found to be effective in reducing pain in the knee (SMD: −0.48 CI: −0.58~−0.37, I^2^: 48%) and hip (SMD: −0.52 CI: −0.97~−0.08). There was no significant difference between the subgroups by joint (*p* = 0.84).

Regarding the effect of intervention duration on strength, it was found to be effective in increasing strength for the duration of 4 weeks or less (SMD: 0.35, CI: 0.07~0.64, I^2^: 0%), 5~8 weeks (SMD: 0.42 CI: 0.34~0.51, I^2^: 0%), and 9 weeks or more (SMD: 0.3 CI: 0.13~0.47, I^2^: 11%). There was no significant difference between the subgroups by the intervention duration (*p* = 0.42). Regarding joint-specific effects, it was found to be effective in increasing strength of the knee (SMD: 0.38, CI: 0.31~0.46, I^2^: 0%) and hip (SMD: 0.65, CI: 0.30~1.00, I^2^: 0%). There was no significant difference between the subgroups by joint (*p* = 0.15).

Regarding the effect of intervention duration on function, no significant effect was observed for 4 weeks or less (SMD: −0.26, CI: −0.55~0.03, I^2^: 0%), but it was found to be effective in reducing functional disability for the duration of 5~8 weeks (SMD: −0.58, CI: −0.73~−0.42, I^2^: 47%) and 9 weeks or more (SMD: −0.58, CI: −0.69~0.47, I^2^: 5%). There was no significant difference between the subgroups by the intervention duration (*p* = 0.13). Regarding joint-specific effects, it was all found to be effective in reducing functional disability of the knee (SMD: −0.57, CI: −0.66~−0.47, I^2^: 33%) and hip (SMD: −0.52, CI: −0.74~−0.30, I^2^: 0%). There was no significant difference between the subgroups by joint (*p* = 0.69).

#### 3.4.5. Publication Bias

To check for publication bias, we visually analyzed the results using a funnel plot (provided in Supplementary Figure S4). Egger’s regression analysis was performed because it was determined that there was asymmetry in the analysis results, and it was determined that there was a risk of publication bias because the *p* values for pain (*p* = 0.03) and function (*p* = 0.03) were less than 0.05 (Table 2). Therefore, after adjusting for error through trim-and-fill, 17 studies were added for function and 14 studies were added for pain (provided in Supplementary Figure S5). The results of the trim-and-fill analysis showed that both pain (SMD: −0.31, CI: −0.43~−0.18) and function (SMD: −0.43, CI: −0.53~−0.33) had significant effects.

## 4. Discussion

This systematic review and meta-analysis showed that resistance training is effective in improving pain, strength, and function in OA. In addition, in the subgroup analysis, pain and strength were effective in all durations during the intervention durations, but function was not effective in less than 4 weeks. In joint-specific comparisons, both knee and hip OA were effective in improving pain, strength, and function.

In this review, both pain and function showed significant effects compared to other groups, but muscle strength showed no effect compared to the usual care group. Regarding the effect of resistance training on OA, Li et al. [15] reported in a meta-analysis that resistance training was effective in improving pain and function compared to the control group. In addition, resistance training has been shown to increase strength and function and decrease pain in older adults with OA [49] and has benefits in increasing knee extensor strength [50,51]. The quadriceps muscle provides stability to the knee joint together with other lower extremity muscles and ligaments, and weakening of the quadriceps muscle increases the load on the passive components of the knee joint, increasing joint stiffness [52]. Increasing quadriceps muscle strength through resistance training reduces the risk of cartilage loss and space narrowing of the tibiofemoral joint [52]. Therefore, it is thought that increased muscle strength through resistance training helps improve pain and function by increasing load absorption and stability of the knee joint [53]. Another reason may be that patients felt their pain was improved on their own due to the placebo effect of resistance training. Contextual effects in chronic pain disorders may be regression to the mean or placebo effects [54]. Messier et al. [37] explained the lack of difference in pain improvement between the high-intensity resistance training group and the control group as the placebo effect. Because the functions and pain measures collected in this review were all patient-reported outcomes, the observed effects cannot be ruled out as a placebo effect.

In addition, Marriott et al. [16] reported that there were differences in the effects on pain and function depending on the comparison group, with the greatest effect compared to no intervention and no effect compared to other exercise or non-exercise (or combined) interventions. In this review, function appeared to be most effective in the no intervention group compared to other comparison groups, but there were no significant differences between groups in pain and strength. The reason for this difference in effect is that this study collected all outcomes, whereas the previous study collected only one outcome when there were two or more outcomes in a study. In addition, this study excluded cases where resistance or strength training was included in the comparison group, but previous studies compared cases by including resistance training. Therefore, it appears that the difference in effect is due to differences in the selection criteria for the comparison group and outcome, and the number of subjects included in the review.

In this review, pain and muscle strength showed effects at all intervention durations, but function showed no effect at durations less than 4 weeks. Marriott et al. [55] reported that resistance training for 3 to 6 months was most effective for pain and physical function than for 12 months or less than 3 months. In contrast, Turner et al. [56] reported that the largest effect sizes for improvements in pain and function occurred at 24 resistance training sessions and 8 to 12 weeks. These conflicting results appear to be mainly due to the fact that OA is influenced by several factors, including pain sensitivity, psychological distress, BMI, muscle strength, inflammation, obesity, and gender [57]. In addition, most of the measures included in this review were conducted over 8 weeks, and there are few studies that measured for 4 weeks or less, so there may be differences in effectiveness.

In this review, the effects of joint-specific resistance training were shown to be effective for both knee OA and hip OA. In the study by Marriott et al. [16], resistance training was also effective in improving pain and function in both hip and knee OA when compared to conservative intervention. However, this review included only two studies out of 27 that focused on hip OA, so caution is needed when interpreting the results. A review by Zacharias et al. [51] also collected studies on knee and hip OA and found that only one of 40 studies focused on hip OA. This suggests that further research on hip OA should be conducted, as there is a paucity of studies focusing on hip OA.

In particular, this review found no heterogeneity in the moderate effect on strength improvements, despite the fact that the studies included all resistance training methods, including aquatic resistance, concentric or eccentric resistance, and fast or slow velocity contractions. In the case of resistance training, the cost of exercise equipment or participation in related programs can be significant, and adjusting the intensity of training to the patient’s condition can increase the patient’s performance and confidence in exercise [3]. This allows patients to choose a resistance training method based on their preference or economic considerations to improve strength. In addition, increases in lower extremity muscle strength are expected to have a positive effect on pain and function [16,50]. Resistance exercise can alleviate OA symptoms by increasing muscle strength, improving articular surface loading, and rebalancing the activation patterns of leg muscles [3]. These findings suggest that resistance training is necessary for OA patients. However, the effect sizes of resistance training in this review were generally moderate. A recent network meta-analysis targeting OA showed that various exercises, including aquatic exercise, yoga, and cycling, in addition to resistance training, were effective [58]. The OARIS guidelines also recommend several types of exercise rather than just one type of exercise [8]. Therefore, there is a need to find more effective combined exercise interventions when used in conjunction with resistance training for OA.

Limitations of this review include the exclusion of surgical patients, which may have led to more positive results, as patients with more severe symptoms were not included in the study. In addition, this review did not include an analysis of the relationship between improvement in outcome and various factors such as age, sex, BMI, and disease severity. For example, women have weaker quadriceps muscles than men and are more prone to reduced knee joint protection [52]. Inflammation plays an important role in the development and progression of OA, and obesity disrupts immune homeostasis and induces inflammation [59]. Because the factors related to OA are extremely diverse, future research needs to be conducted that considers the relationship between various factors and improvement in outcome. In terms of methodological limitations, most of the included studies were not blinded to patients and therapists, and more than half of the studies did not perform random allocation and intention-to-treat analysis, which may lead to bias and overestimation in the results.

## 5. Conclusions

Resistance training was effective in improving OA pain and function compared to all groups, but had no effect on muscle strength compared to the usual care group. The effects on pain and strength appeared to be beneficial regardless of the intervention duration, while function appeared to be ineffective for durations of less than 4 weeks. In terms of joint-specific effects, resistance training was effective for both knee OA and hip OA. In particular, low heterogeneity in strength gains was observed despite the inclusion of various resistance training methods. Patients can choose the desired resistance training method to improve strength, taking into account their preferences and economic situation. In the future, research needs to be conducted that considers the relationship between various factors related to OA and improvement in outcome.

## Figures and Tables

**Table 1:** Subgroup analysis.

Outcome Measure	Subgroup		The Number of Participants (EG/CG)	Effects Size [SMD, 95% CI]	I^2^ (%)	Test for Subgroup Differences
Pain	Duration	~4 weeks	112/112	−0.42 [−0.78~−0.06]	38%	*p* = 0.95
		5~8 weeks	508/492	−0.51 [−0.64~−0.38]	0%	
		9 weeks~	841/821	−0.47 [−0.64~−0.30]	61%	
	Joint	Knee	1421/1385	−0.48 [−0.58~−0.37]	48%	*p* = 0.84
		Hip	40/40	−0.52 [−0.97~−0.08]	-	
Strength	Duration	~4 weeks	96/96	0.35 [0.07~0.64]	0%	*p* = 0.42
		5~8 weeks	1159/1143	0.42 [0.34~0.51]	0%	
		9 weeks~	331/317	0.3 [0.13~0.47]	11%	
	Joint	Knee	1516/1492	0.38 [0.31~0.46]	0%	*p* = 0.15
		Hip	70/64	0.65 [0.30~1.00]	0%	
Function	Duration	~4 weeks	96/96	−0.26 [−0.55~0.03]	0%	*p* = 0.13
		5~8 weeks	718/693	−0.58 [−0.73~−0.42]	47%	
		9 weeks~	831/798	−0.58 [−0.69~−0.47]	5%	
	Joint	Knee	1485/1427	−0.57 [−0.66~−0.47]	33%	*p* = 0.69
		Hip	160/160	−0.52 [−0.74~−0.30]	0%	

**Table 2:** Egger’s regression.

Outcome	t	df	*p*-Value
Pain	−2.23	58	*p* = 0.03
Strength	−0.11	52	*p* = 0.92
Function	−2.27	73	*p* = 0.03

## Data Availability

Data can be requested from the corresponding author and will be released on reasonable request.

## Supplementary Materials

The following supporting information can be downloaded at: https://www.mdpi.com/article/10.3390/jpm14121130/s1, Table S1: Search strategy; Table S2: General characteristics; Table S3: PEDro scale; Figure S1: Forest plot pain; Figure S2: Forest plot strength; Figure S3: Forest plot function; Figure S4: Funnel plot; Figure S5: Trim and fill.
