# Supplementary material for: The Effects of Resistance Training on Pain, Strength, and Function in Osteoarthritis: Systematic Review and Meta-Analysis
Source: J Pers Med. 2024 Nov 30;14(12):1130. doi: 10.3390/jpm14121130 (PMC11676110; doi:10.3390/jpm14121130)
Supplement: Supplementary file 1 [file jpm-14-01130-s001.zip › Supplementary Table S1 Search strategy.pdf]

Database	Search strategy
PubMed	#1 "Osteoarthritis"[MeSH Terms]<br>#2: Osteoarthritis<br>#3 "Knee Joint"[MeSH Terms]<br>#4 Knee<br>#5 "Hip Joint"[MeSH Terms]<br>#6 Hip<br>#7 "Resistance Training"[MeSH Terms]<br>#8 Resistance training<br>#9 Strength training<br>#10 #1 OR #2<br>#11 #3 OR #4 OR #5 OR #6<br>#12 #7 OR #8 OR #9<br>#13 #10 AND #11 AND #12
CINAHL	S1 (MH "Osteoarthritis+")<br>S2 (osteoarthr*)<br>S3 (MH “Knee”)<br>S4 (knee*)<br>S5 (MH “Hip”)<br>S6 (Hip*)<br>S7 (MH “resistance training”)<br>S8 (resistance training*)<br>S9 (strength training*)<br>S10 S1 OR S2<br>S11 S3 OR S4 OR S5 OR S6<br>S12 S7 OR S8 OR S9<br>S13 S10 AND S11 AND S12
Cochrane Library	#1 MeSH descriptor: [Osteoarthritis] explode all trees<br>#2 osteoarthr*<br>#3 (#1 OR #2)<br>#4 MeSH descriptor: [Knee] explode all trees<br>#5 knee*<br>#6 MeSH descriptor: [Hip] explode all trees<br>#7 Hip*<br>#8 (#4 OR #5 OR #6 OR #7)<br>#9 MeSH descriptor: [Resistance Training] explode all trees<br>#10 Resistance training*<br>#11 strength training*<br>#12 (#9 OR #10 OR #11)<br>#13 (#3 AND #8 AND #12)
EMBase	#1 'osteoarthritis'/exp<br>#2 'osteoarthritis'<br>#3 'knee'/exp<br>#4 'knee'<br>#5 'hip'/exp<br>#6 'hip'<br>#7 'resistance training'/exp<br>#8 'resistance training'<br>#9 'strength training'<br>#10 #1 OR #2<br>#11 #3 OR #4 OR #5 OR #6<br>#12 #7 OR #8 OR #9

	#13 #10 AND #11 AND #12
