# Supplementary material for: The Effects of Resistance Training on Pain, Strength, and Function in Osteoarthritis: Systematic Review and Meta-Analysis
Source: J Pers Med. 2024 Nov 30;14(12):1130. doi: 10.3390/jpm14121130 (PMC11676110; doi:10.3390/jpm14121130)
Supplement: Supplementary file 1 [file jpm-14-01130-s001.zip › Supplementary Table S2 General characteristics.pdf]

Supplementary Table S2. General characteristics of the selected studies

Study	Participants: Male/Female Age: M(SD), BMI: M(SD)		Joint	Radiodiagnosis: Grade - EG/CG	Intervention			Outcome
	EG	CG			EG	CG	Session	
Azizi, 2019 [20]	15/0<br>63.5(4.7)<br>-	16/0<br>65.5(3.3)<br>-	Knee	None	Strength training in aquatic exercise program: Knee extensor	No treatment	1 hour 3 times per week for 8 weeks	VAS
Beckwée, 2017 [21]	6/11<br>63.7(8.08)<br>28(4.68)	11/7<br>60.1(9.48)<br>28.0(3.07)	Knee	1&2: 12/13<br>3&4: 5/5	Strength training: Knee extensors, hamstring, hip abductor, and hip adductor muscles	Walking training	40-45 minutes 3 times per week for 18 weeks	ICOAP:<br>Constant pain, intermittent pain, total pain
Bieler, 2022 [22]	4/11<br>67.1(3.9)<br>25.3(3.9)	4/8<br>69.1(5.1)<br>26.3(4.9)	Hip	0: 4/6<br>1: 2/1<br>2: 1/1<br>3: 4/3<br>4: 4/1	Resistance training: Leg press, seated knee extension, hip extension in standing position	Nordic walking	1 hour 3 times per week for 4 months	QMVC (Nm)<br>LEP (W)
Bruce-Brand, 2012 [23]	6/4<br>63.4(5.9)<br>33.9(8.3)	3/3<br>65.2(3.1)<br>31.7(4.1)	Knee	3~4	Resistance training: Knee presses, bottle knee presses, extended leg raises, leg extensions, wall squats and hamstring curls	Standard care	30 minutes 3 times per week for 6 weeks	WOMAC: Physical function, pain, stiffness<br><br>Nm: Isometric/isokinetic peak torque
Chang, 2012 [24]	0/24<br>65(8.4)<br>24.9(3.3)	0/17<br>70.8(8.4)<br>25.7(3.6)	Knee	2: 9/3<br>3: 15/14	Elastic-band exercise: Leg press	Conventional modality treatments	2 times per week for 8 weeks	WOMAC: Pain, stiffness, physical function
de Almeida, 2020 [25]	5/16<br>55.2(7.4)<br>26(3.14)	4/16<br>53.8(7.7)<br>27(2.7)	Knee	2: 15/15<br>3: 6/5	conventional strength training: Quadriceps, hamstrings, hip adductors and abductors isometric muscle contraction	Educational protocol	3 times per week for 14 weeks	Knee pain, knee extension MIVC

de Almeida, 2021 [26]	5/16<br>55.2(7.4)<br>26(3.14)	4/16<br>53.8(7.7)<br>27(2.7)	Knee	2: 15/15<br>3: 6/5	Resistance exercises: Quadriceps, hamstring, hip abductors, hip adductors, and trunk	Educational protocol	3 times per week for 14 weeks	WOMAC Pain, stiffness, function, total
DeVita, 2018 [27]	7/8<br>56.2(8.9)<br>27.9(3.9)	5/10<br>58.1(6.5)<br>26.4(4.0)	Knee	1: 3/2<br>2: 1/4<br>3: 8/7<br>4: 3/2	Strength training: Leg extension, leg press and forward lunge exercises	Control group	3 times per week for 12 weeks	WOMAC: Pain, function, total<br><br>Nm/kg: Isokinetic quadriceps muscle strength, Maximum internal knee extensor torque<br>N/kg: Maximum knee compressive/quadriceps force<br>W/kg: Maximum positive/negative quadriceps power<br>J/kg: Quadriceps positive/negative work
Farr, 2010 [28]	F - 73%<br>55.5(7.3)<br>27.5(4.5)	F - 72%<br>55.8(6.1)<br>28.0(4.0)	Knee	2: 109	Resistance training: Leg press, leg curl, hip abduction and adduction, straight leg lift, incline dumbbell press, seated row, and calf raise	Self-management	1 hour 3 times per week for 9 months	WOMAC: Pain
Hermann, 2016 [29]	13/27<br>70.0(7.7)<br>28.2(5.3)	15/25<br>70.8(7.5)<br>27.4(3.8)	Hip	none	Explosive-type resistance training: Hip extension, knee extension, knee flexion and leg press in a seated position	Care as usual	1 hour 2 times per week for 10 weeks	HOOS: ADL, pain, symptoms, sports & recreation, QOL<br><br>Leg extension power

Isaramalai, 2018 [30]	EG1: 3/22 68.0(5.80) 23.9(4.60)<br><br>EG2: 9/16 66.9(4.50) 26.3(4.10)	5/20 63.7(4.30) 24.7(4.00)	Knee	EG1/EG2/CG 1: 11/4/7 2: 5/9/8 3: 9/12/10	EG1: participatory ergonomic management with non-weight-bearing exercise (PEM-NWE)<br><br>EG2: participatory ergonomic management with progressive resistance exercise (PEM-PRE)<br><br>: Straight-leg raise, Hip abduction/flexion/extension, Knee flexion, straight-leg raise, quad set	CG: Standard treatment	3 times per week for 8 weeks	WOMAC: Total, pain, stiffness, physical function
Jan, 2008 [31]	EG1 7/27 63.3(6.6) -<br><br>EG2 7/27 61.8(7.1) -	CG1 5/25 62.8(6.3) -	Knee	EG1/EG2/CG 1: 8/9/7 2: 43/43/40 3: 17/16/13	EG1: High-resistance exercise<br><br>EG2: Low-resistance exercise<br><br>: Leg press	No exercise	30~50 minutes 3 times per week for 8 weeks	WOMAC: Pain, function<br><br>Knee extension/flexion torque: 60/120/180 degrees
Jorge, 2015 [32]	0/29 61.7(6.4) 30.6(5.75)	0/31 59.9(7.5) 31.4(4.42)	Knee	Grade 1:2 EG: 24.1:31.0 CG: 38.7:22.6	Progressive resistance exercise: Knee extension/ flexion and hip abduction/adduction	No treatment	2 times per week for 12 weeks	VAS, WOMAC: pain, stiffness, function, total SF-36: function, role limitation
Kuş, 2023 [33]	7/17 60.04(6.05) 29.59(3.77)	8/16 58.54(7.05) 30.53(3.86)	Knee	2: 10/5 3: 14/19	Progressive resistance exercises: Knee extension/flexion, hip abduction/adduction	Balance exercises	45 minutes 3 times per week for 8 weeks	VAS: activity, rest, night<br>WOMAC<br>Knee flexion, extension<br>Hip abduction, adduction

								SF-36: physical function, pain
Lin, 2009 [34]	12/24<br>61.6(7.2)<br>-	CG1:<br>11/25<br>63.7(8.2)<br><br>CG2:<br>10/26<br>62.2(6.7)	Knee	EG/CG1/CG2<br>2: 15/16/17<br>3: 21/20/19	Strength training: Knee flexion/extension	CG1: Computer game foot-stepping exercise<br><br>CG2: No treatment	3 times per week for 8 weeks	WOMAC: pain, function
Lin, 2007 [35]	5/21<br>61.0(7.7)<br>-	CG1:<br>9/20<br>61.6(8.1)<br>-<br><br>CG2:<br>5/21<br>62.8(6.3)<br>-	Knee	EG/CG1/CG2<br>1: 7/10/5<br>2: 35/39/39<br>3: 10/9/8	Closed kinetic chain knee exercise: Knee flexion/extension	CG1: computerized proprioception facilitation exercise<br><br>CG2: no treatment	3 times per week for 8 weeks	Knee flexion/extension: 60/120/180 degrees
Messier, 2021 [36]	EG1:<br>75/52<br>67(9)<br>31(6)<br><br>EG2:<br>75/51<br>64(8)<br>31(6)	76.48<br>64(7)<br>32(5)	Knee	2~3	EG1: High intensity<br><br>EG2: Low intensity<br><br>: Hip abduction and adduction; leg curl, leg extension, and leg press; and seated calf, compound row, vertical chest, lower back, and abdomen.	Attention-control group	60 minutes 3 times per week for 18 months	WOMAC: Pain
Oh, 2021 [37]	-<br>71.05(6.45)<br>24.67(2.59)	-<br>70.55(4.80)<br>26.04(4.56)	Knee	>3	Self-directed home-based resistance training: Spread knees, knee extension, leg press, glute bridge, sit-up,	Health education program	2~3 times per week for 5 months	WOMAC: Total, pain, stiffness, physical function

					AB crunch with twist, leg raise, hip adduction, superman exercise			
Pazit, 2018 [38]	5/4<br>67.78(6.2)<br>28.2(5.6)	4/5<br>70.44(7.8)<br>28.4(3.9)	Knee	None	High speed resistance training: Leg press, sit to stand, squat, step-up, calf raises lunges, going up stairs	No intervention	2 times per week for 8 weeks	WOMAC: Total, Pain, function, stiffness, 3RM (leg press)
Rogers, 2012 [39]	Female 70%<br>70.8(6.5)<br>28.2	CG1:<br>Female: 69%<br>70.7 (10.7)<br>28.9<br><br>CG2:<br>Female: 67%<br>71.2(10.9)<br>30.8	Knee	None	Elastic resistance bands training: Ankle extension/flexion, knee extension/flexion, hip abduction/adduction/internal rotation/external rotation, leg press* (hip and knee extension), hip hyper-extension	CG1: Kinesthesia, balance and agility exercise training<br><br>CG2: Usual care	30~40 minutes 3 times per week for 8 weeks	WOMAC: Total, Pain, stiffness, function
Samut, 2015 [40]	62.46(7.71)<br>30.54(4.45)	CG1<br>57.57(5.79)<br>33.94(7.33)<br><br>CG2<br>60.92(8.85)<br>30.36(5,67)	Knee	2~3	Isokinetic exercise: Knee flexion/extension	CG1: Aerobic exercise<br><br>CG2: No intervention	3 times per week for 6 weeks	VAS, WOMAC: Pain, stiffness, function, total
Sayers, 2012 [41]	EG1<br>3/9<br>66.9(4.9)<br>28.4(5.7)<br><br>EG2<br>2/8<br>65.9(8.3)<br>33.1(8.9)	3/8<br>68.4(8.1)<br>30.8(6.8)	Knee	EG1/EG2/CG<br>Mean (SD)<br>Right - 1.5(0.8)/ 1.9(0.9)/ 2.0(0.9)	EG1: High speed movement resistance training<br><br>EG2: Slow speed movement<br><br>: Knee extension	Assisted stretching exercise	3 times per week for 12 weeks	WOMAC: Pain, function

				Left - 1.6(0.5)/ 1.3(1.00/ 1.5(1.0)				
Vincent, 2023 [42]	EG1: 9/18 69.5(6.5) 32.8(7.4)<br><br>EG2: 9/21 66.8(5.4) 28.7(6.6)	7/21 68.6(7.1) 31(6.2)	Knee	2-3	EG1: concentric resistance training<br><br>EG2: eccentric resistance training<br><br>: Leg press, knee extension/flexion, chest press, seated row, shoulder press, biceps curl and calf press.	No intervention	2 times per week for 16 weeks	Pain: 6WMT, treadmill exercise
Vincent, 2019 [43]	EG1 Female: 67% 69.5(6.5) 32.8(7.4)<br><br>EG2 Female: 70% 66.8(5.4) 28.7(6.6)	Female: 66% 68.6(7.2) 30.1(6.2)	Knee	2~3	EG1: Concentric resistance training<br><br>EG2: Eccentric resistance training<br><br>: Leg press, knee flexion/extension, chest press, seated row, overhead press, biceps curl, and calf press.	Non-exercise	2 times per week for 4 months	Chest press, Seated row, shoulder press
Vincent, 2020 [44]	EG1: Women: 66.7% 69.5(6.5) 32.8(7.3)<br><br>EG2:	Women: 65.6% 68.6(7.1) 32.8(18.2)	Knee	2~4	EG1: concentric resistance training<br><br>EG2: eccentric resistance training	Normal activities	2 times per week for 4 months	Pain: Chair rise pain, stair climb, 6MWK

	Women: 70%<br>66.8(5.4)<br>28.7(6.6)				: Leg press, knee flexion/extension, calf press, chest press, seated row, shoulder press, and biceps curl.			
Waller, 2017 [45]	0/43<br>63.8(2.4)<br>26.6(3.8).	0/44<br>63.9(2.4)<br>27.1(3.5)	Knee	1: 23/24<br>2: 20/20	Aquatic Resistance training: knee flexion/extension, kickback, hip abduction/adduction/flexion/extension	Usual care	1 hour 3 times per week for 16 weeks	KOOS: Pain, symptoms, ADL, sport, QOL
Wortley, 2013 [46]	4/9<br>69.5(6.7)<br>30.5(6.0)	2/4<br>70.5(5.0)<br>30.0(6.2)	Knee	Expressed in median (range)<br>EG - 2(2)<br>CG - 2(1)	Open kinetic chain resistance training program: Leg extension, hamstring curl, straight leg raise, hip abduction/adduction/flexion, calf raise.	Usual physical activity	1 hour 2 times per week for 10 weeks	WOMAC: Pain, stiffness, function

EG: Experimental group, CG: Control group, BMI: Body mass index, VAS: Visual analog scale, WOMAC: Western Ontario and McMaster Universities arthritis index, ICOAP: Intermittent and constant osteoarthritis pain, HOOS: hip disability and osteoarthritis outcome score, KOOS: knee injury and osteoarthritis outcome score, SF-36: 36-item short form survey, QMVC Quadriceps maximal isometric voluntary contraction, LEP: leg extensor power, QOL: Quality of life, 6MWK: 6-min walk ADL: Activities of daily living
