# Supplementary material for: The Effects of Resistance Training on Pain, Strength, and Function in Osteoarthritis: Systematic Review and Meta-Analysis
Source: J Pers Med. 2024 Nov 30;14(12):1130. doi: 10.3390/jpm14121130 (PMC11676110; doi:10.3390/jpm14121130)
Supplement: Supplementary file 1 [file jpm-14-01130-s001.zip › Supplementary Table S3 PEDro scale.pdf]

Supplementary Table S3. Quality assessment with PEDro scale

Study	1	2	3	4	5	6	7	8	9	10	11	Total
Azizi, 2019 [20]	-	O	X	O	X	X	O	O	O	O	O	7
Beckwée, 2017 [21]	-	O	O	O	X	X	O	X	X	O	O	6
Bieler, 2022 [22]	-	O	X	O	X	X	O	X	X	O	O	5
Bruce-Brand, 2012 [23]	-	O	X	O	X	X	O	X	X	O	O	5
Chang, 2012 [24]	-	O	X	O	O	X	O	O	X	O	O	7
de Almeida, 2020 [25]	-	O	O	O	X	X	O	O	X	O	O	7
de Almeida, 2021 [26]	-	O	X	O	X	X	O	O	X	O	O	6
DeVita, 2018 [27]	-	O	X	O	X	X	X	O	X	O	O	5
Farr, 2010 [28]	-	O	O	O	X	X	X	X	X	O	O	5
Hermann, 2016 [29]	-	O	O	O	X	X	X	O	O	O	O	7
Isaramalai, 2018 [30]	-	O	X	O	X	X	X	O	O	O	O	6
Jan, 2008 [31]	-	O	X	O	X	X	O	O	O	O	O	7
Jorge, 2015 [32]	-	O	O	O	X	X	O	O	O	O	O	8
Kuş, 2023 [33]	-	O	O	O	X	X	X	O	X	O	O	6
Lin, 2009 [34]	-	O	O	O	X	X	O	O	O	O	O	8
Lin, 2007 [35]	-	O	X	O	X	O	X	O	X	O	O	6
Messier, 2021 [36]	-	O	X	O	O	X	O	O	X	O	O	7
Oh, 2021 [37]	-	O	X	O	X	X	X	X	O	O	O	5
Pazit, 2018 [38]	-	O	O	O	X	X	X	O	X	O	O	6
Rogers, 2012 [39]	-	O	X	O	O	X	O	X	X	O	O	6
Samut, 2015 [40]	-	O	X	O	X	X	X	O	X	O	O	5
Sayers, 2012 [41]	-	O	X	O	X	X	O	X	O	O	O	6
Vincent, 2023 [42]	-	O	O	O	X	X	O	X	X	O	O	6
Vincent, 2019 [43]	-	O	O	O	X	X	O	X	O	O	O	7
Vincent, 2020 [44]	-	O	O	O	X	O	X	X	O	O	O	7
Waller, 2017 [45]	-	O	O	O	X	X	X	O	O	O	O	7
Wortley, 2013 [46]	-	O	X	O	X	X	X	X	X	O	O	4

1: Eligibility criteria specified 2. Randomized subjects 3: Allocation concealed 4: similar at baseline 5: Subjects blinding 6: Therapists blinding 7: assessors blinding 8: Outcome measures for 85% or more of participants 9: Intention-to-treat 10: Reported statistical comparisons between groups 11: Provide point measures or measures of variability
