# Supplementary figures and images for: The Effects of Resistance Training on Pain, Strength, and Function in Osteoarthritis: Systematic Review and Meta-Analysis
Source: J Pers Med. 2024 Nov 30;14(12):1130. doi: 10.3390/jpm14121130 (PMC11676110; doi:10.3390/jpm14121130)

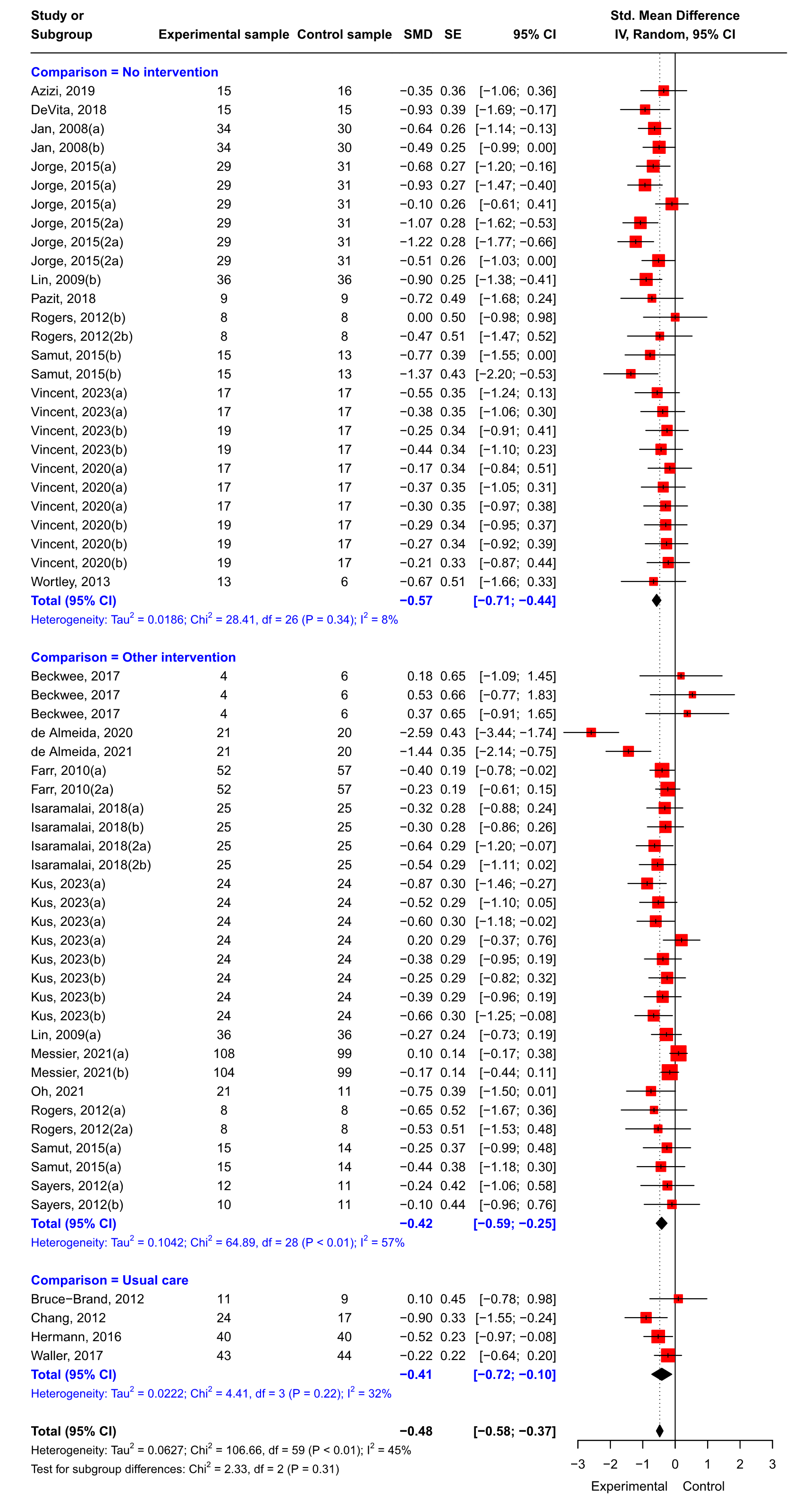

Supplement: Supplementary file 1 [file jpm-14-01130-s001.zip › Supplementary Figure S1 Forest plot pain.png]

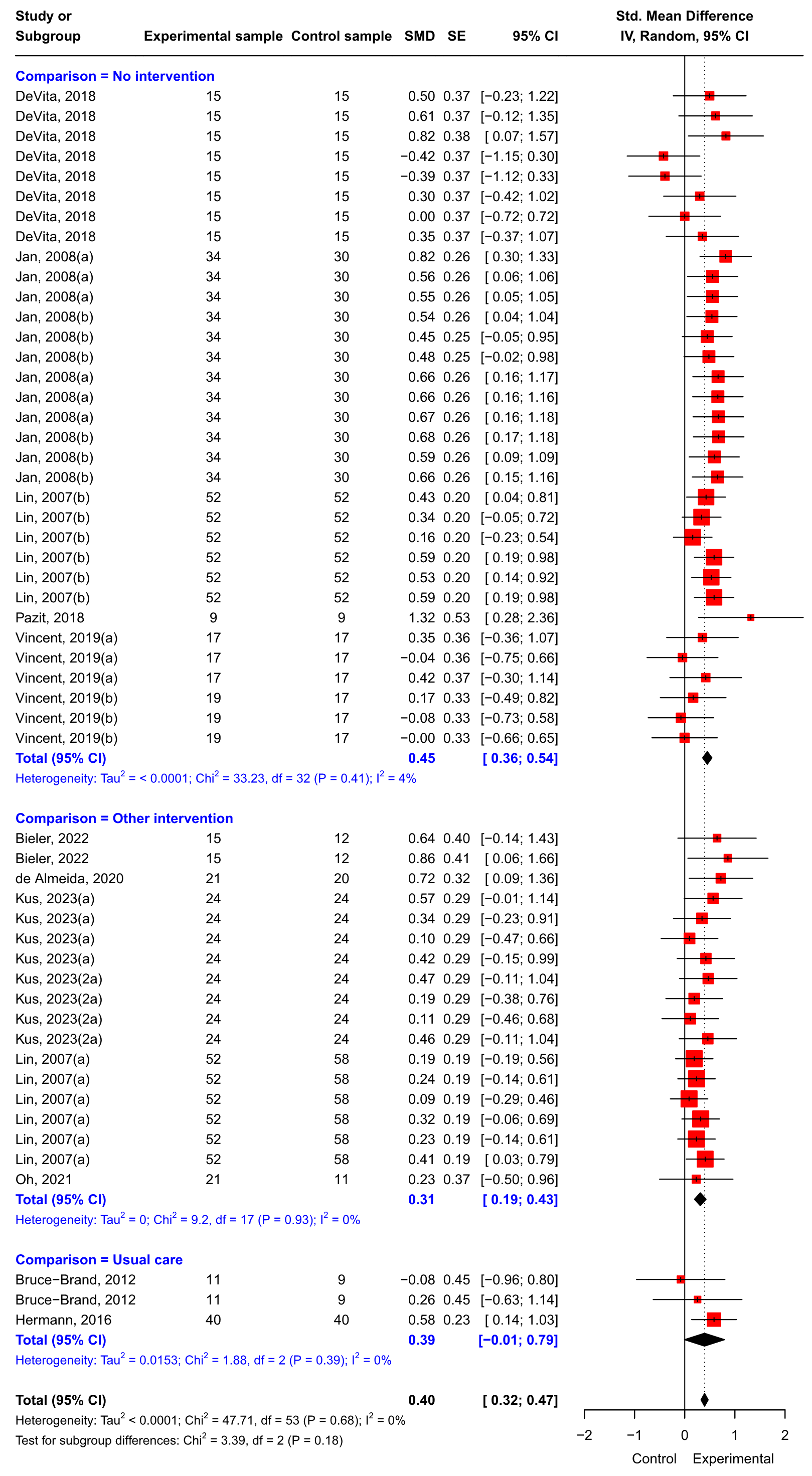

Supplement: Supplementary file 1 [file jpm-14-01130-s001.zip › Supplementary Figure S2 Forest plot strength.png]

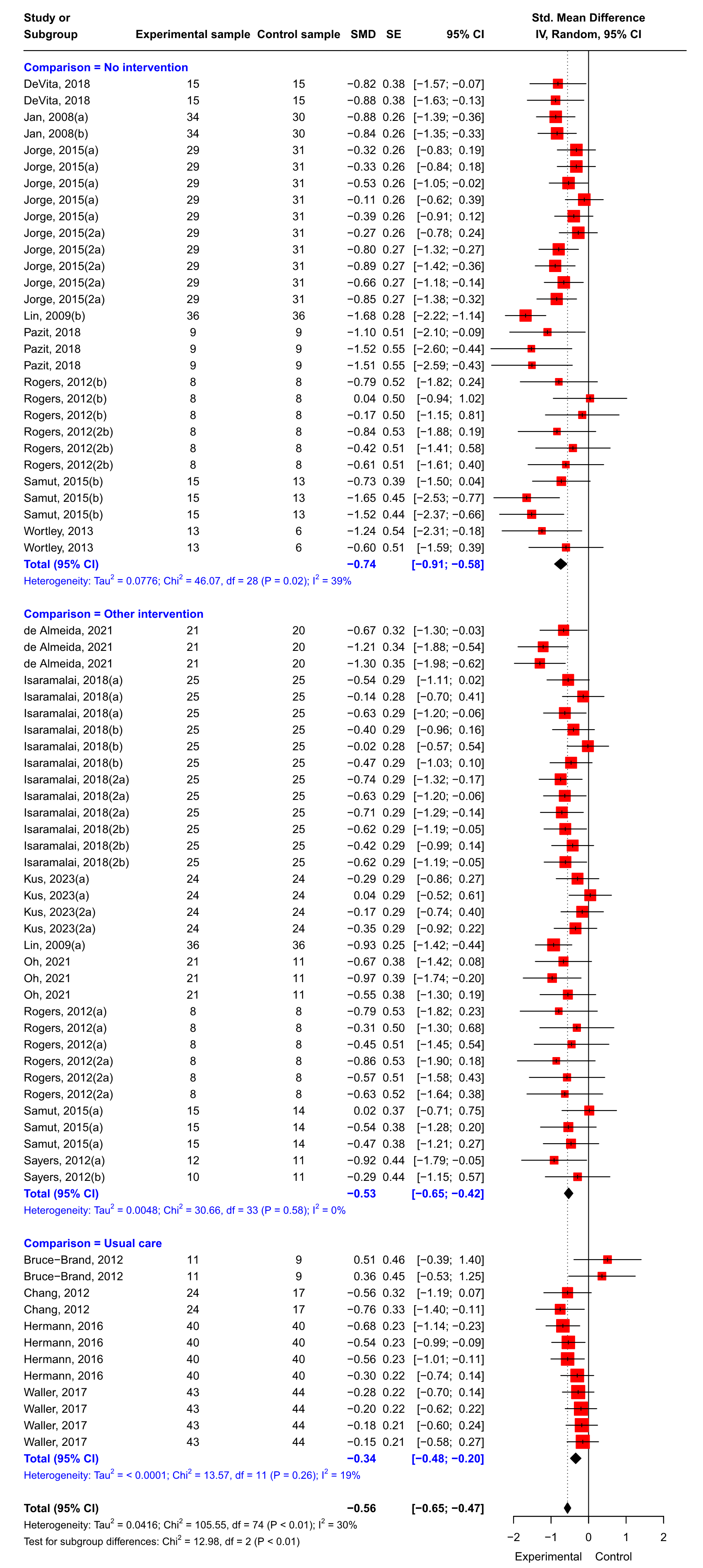

Supplement: Supplementary file 1 [file jpm-14-01130-s001.zip › Supplementary Figure S3 Forest plot function.png]

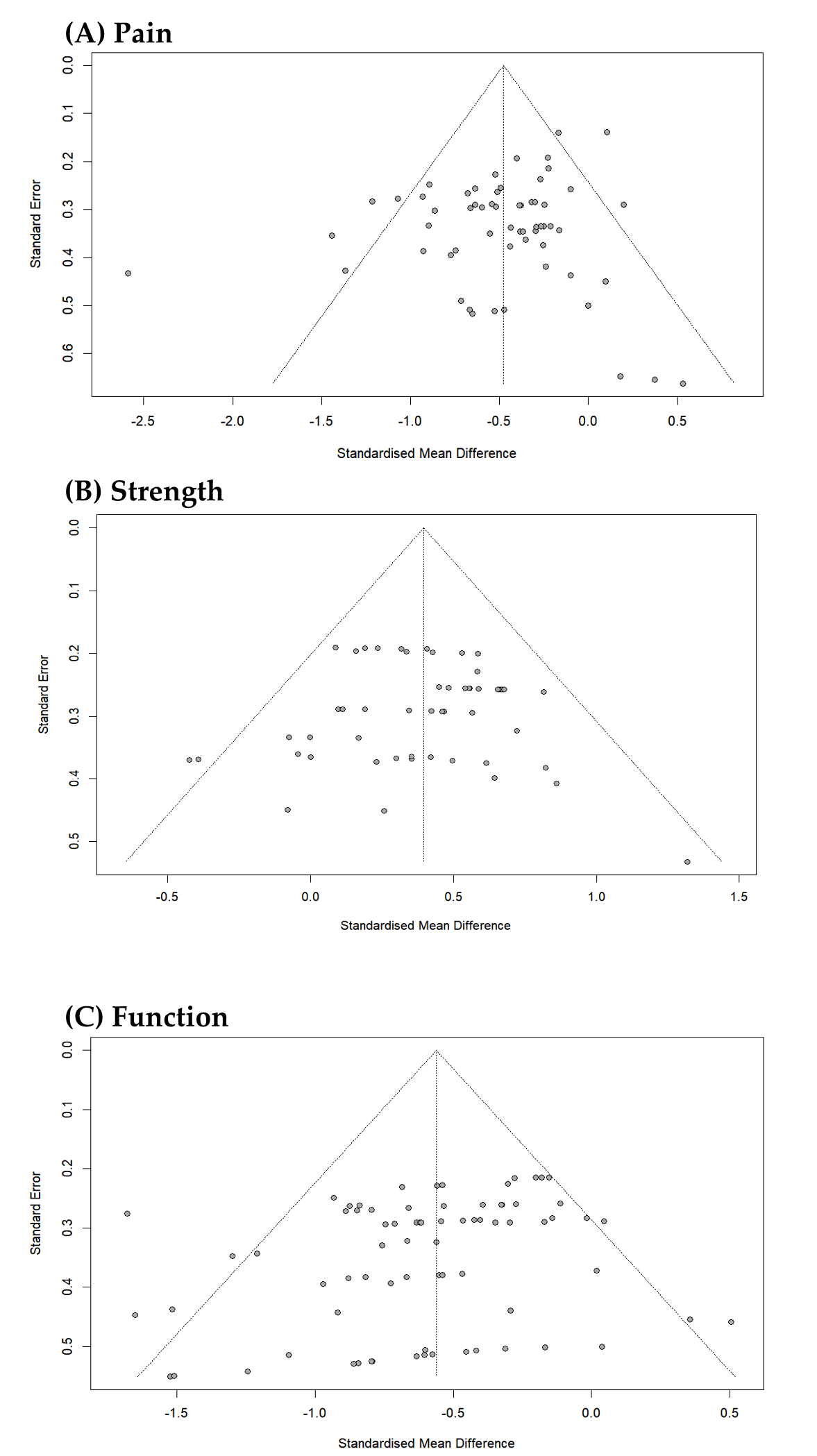

Supplement: Supplementary file 1 [file jpm-14-01130-s001.zip › Supplementary Figure S4 Funnel plot.png]

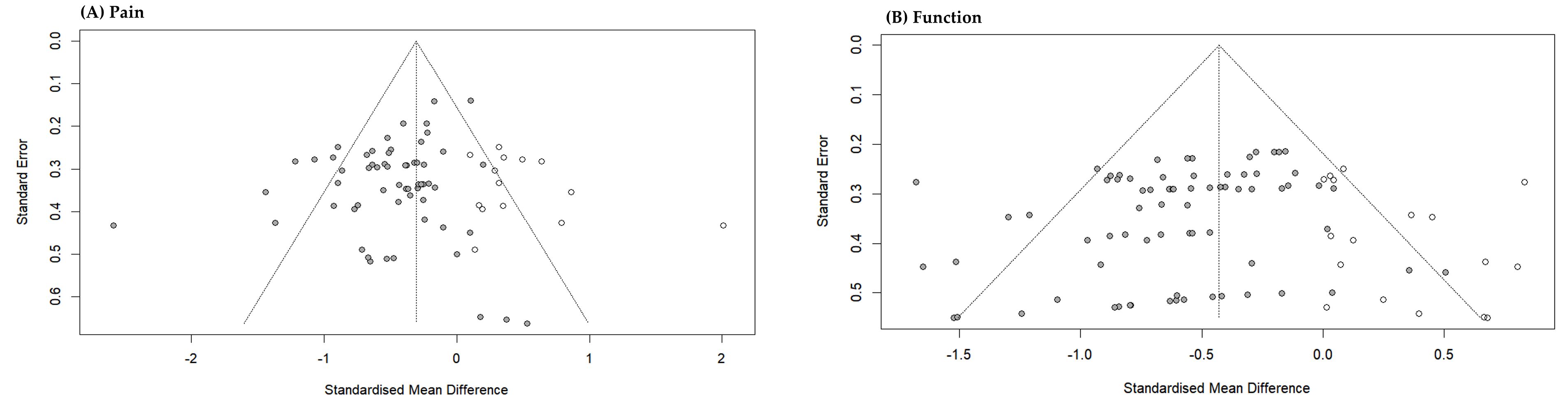

Supplement: Supplementary file 1 [file jpm-14-01130-s001.zip › Supplementary Figure S5 Trim and fill.png]
